# Risk factors for secondary fractures to percutaneous vertebroplasty for osteoporotic vertebral compression fractures: a systematic review

**DOI:** 10.1186/s13018-021-02722-w

**Published:** 2021-10-30

**Authors:** Wei Mao, Fei Dong, Guowei Huang, Peiliang He, Huan Chen, Shengnan Qin, Aiguo Li

**Affiliations:** 1 Guangzhou Institute of Traumatic Surgery, Department of Orthopedics, Guangzhou Red Cross Hospital, Medical College, Jinan University, Guangzhou, China; 2 Department of Clinical Medicine, Guizhou Medical University, Guiyang, China

**Keywords:** Vertebral compression fractures (VCFs), Percutaneous vertebroplasty, Risk factor, Meta-analysis

## Abstract

**Background:**

Osteoporotic vertebral compression fracture (OVCF) is one of the most common fragile fractures, and percutaneous vertebroplasty provides considerable long-term benefits. At the same time, there are many reports of postoperative complications, among which fracture after percutaneous vertebroplasty is one of the complications after vertebroplasty (PVP). Although there are many reports on the risk factors of secondary fracture after PVP at home and abroad, there is no systematic analysis on the related factors of secondary fracture after PVP.

**Methods:**

The databases, such as CNKI, Wan Fang Database and PubMed, were searched for documents on secondary fractures after percutaneous vertebroplasty published at home and abroad from January 2011 to March 2021. After strictly evaluating the quality of the included studies and extracting data, a meta-analysis was conducted by using Revman 5.3 software.

**Results:**

A total of 9 articles were included, involving a total of 1882 patients, 340 of them diagnosed as secondary fractures after percutaneous vertebroplasty.

**Conclusion:**

The additional history of fracture, age, bone mineral density (BMD), bone cement leakage, intravertebral fracture clefts and Cobb Angle might be risk factors related to secondary fractures after percutaneous vertebroplasty for osteoporotic vertebral compression fractures. The height of vertebral anterior and body mass index (BMI) were not correlated.

## Introduction

Vertebral compressive fractures (VCFs) are common in elderly populations. VCFs are caused mainly by osteoporosis and can result in back pain, spinal deformities, impaired mobility, reduced pulmonary function, clinical depression, neural compromise, and even paralysis [1–4]. Population studies indicate that the annual incidence of VCFs is 10.7% for women and 5.7% for men [5]. Furthermore, the prevalence of VCFs in those 80 and older is about 30%, while the prevalence in those under 80 is 5–10% [6]. Osteoporotic vertebral compression fracture (OVCF) is one of the most common fragile fractures, with a prevalence of 30–50% in people over 50 years of age [7]. It causes severe pain and disability, raises the risk of secondary fracture more than fourfold [8, 9], and increases the risk of mortality [10]. Percutaneous vertebroplasty provides considerable long-term benefits. At the same time, there are many reports of postoperative complications, among which fracture after percutaneous vertebroplasty is one of the complications after percutaneous vertebroplasty. It has been reported in the literature that 20% of patients are retreated with VCFs within one year of the first fracture [11]. This has become a growing disease and a major health problem worldwide [12, 13], and this will significantly increase the social and economic burden on society and families. There are also many reports on the related factors leading to postoperative refracture, such as the additional history of fracture, age, BMD, bone cement leakage, intravertebral fracture clefts, Cobb angle, the height of vertebral anterior and BMI. Although there are many reports on the risk factors of secondary fracture after PVP at home and abroad, there is no systematic analysis on the related factors of secondary fracture after PVP. With the popularization of translational medicine knowledge, we should make translational medicine establish a two-way flowing marriage between basic disciplines and clinical disciplines. Problems are discovered in clinical practice and then brought to basic research [14]. Through translational medicine, interdisciplinary disciplines can play a greater role, which also brings greater work efficiency to clinical work and greater benefits to patients.

Thus, this study aims to explore the risk factors of secondary fractures after percutaneous vertebroplasty from evidence-based medicine, to provide scientific basis for preventing and reducing the morbidity and mortality of secondary fracture after PVP.

## Materials and methods

This systematic review and meta-analysis were designed in accordance with the preferred reporting items for systematic reviews and meta-analyses (PRISMA) statements [15].

### Data sources and search strategy

A systematic search of the available literature in any language was conducted using China National Knowledge Infrastructure (CNKI), Wan Fang Database, PubMed, PubMed, Cochrane Library and EMBASE from their respective inception to January 2011. We used the following terms and combinations: (“Percutaneous vertebroplasty”) AND (“vertebral compression fractures”) AND (“Secondary fractures”) AND (“Risk factors “OR” Relative risks”) AND (“Cohort studies”).

### Study selection and quality appraisal

Furthermore, the reference lists of all identified studies were searched manually to ensure that all relevant articles were captured. Two researchers independently screened and evaluated the literature quality according to the inclusion and exclusion criteria, and cross-checked repeatedly. When no consensus was reached, the third researcher was consulted, and finally, all the literature that met the inclusion criteria was retrieved. Finally, the uniform data extraction forms were used to extract relevant data. The quality of non-randomized studies was evaluated according to the methods of bias assessment recommended by the Cochrane Collaboration, including selection of case and control groups, comparability between groups, and assessment of exposure. According to the Newcastle–Ottawa Scale (NOS), the quality of the literature that met the inclusion criteria was evaluated. The full score of the NOS scale was 10 points, and the higher the score, the higher the quality of the literature. The score of 8 or above is high quality, the score of seven is high quality, the score of six is medium quality, and the score of 5 or less is low quality.

### Inclusion criteria

The inclusion criteria included as follows: (1) Subjects were recruited from patients suffered from secondary fractures after PVP confirmed by various medical institutions, (2) study designs included retrospective observational studies, (3) Published from January 2014 to November 2019 at home and abroad, and the risk factors involved were the additional history of fracture, age, BMD, bone cement leakage, intravertebral fracture clefts, Cobb Angle, the height of vertebral anterior and BMI and (4) Odds ratio (OR) and 95% confidence interval (CI) were provided in the study results.

### Exclusion criteria

The exclusion criteria were as follows: (1) review articles, case reports, editorials and letters; (2) duplicate articles and/or articles with overlapping patient populations; and (3) data that could not be extracted. (4) Incorrect literature statistical methods and imperfect statistical outcome indicators.

### Data extraction

Data from eligible studies were extracted by two authors independently, and discrepancies were resolved by discussion with a third reviewer. We collected the following information according to pre-defined criteria: (1) general characteristics of studies (first author’s last name, year of publication, study design, Sample size of study subjects, and follow-up duration); (2) Indicators: the Additional history of fracture; Age; BMD; Bone cement leakage; intravertebral fracture clefts; Cobb Angle; the height of vertebral anterior and BMI. When these items were reported insufficiently in eligible articles, we set out to contact the authors to obtain further information. The characteristics of the include studies were listed in Table 1.

**Table 1:** Characteristics of the included studies. Risk factors: 1. Age 2. BMI 3. The additional history of fracture 4. The height of vertebral anterior 5. BMD 6. Bone cement leakage 7. Intravertebral fracture clefts 8. Cobb Angle

Study	Country	Years	Study design	Total events	Secondary fracture events	Age (years)	Follow-up months	Risk factors	NOS score
Bi et al. [16]	China	2017	Retrospective observational studies	177	28	62.5 ± 13.5	15.5 ± 3.2	1, 3, 5	7
Song et al. [17]	China	2018	Retrospective observational studies	93	25	48.6 ± 1.3	12	1, 2, 3, 4	8
Zhou et al. [18]	China	2020	Retrospective observational studies	144	56	69.50 ± 1.30	24–36	1, 3, 4, 5, 6, 8	8
Ji et al. [19]	China	2019	Retrospective observational studies	618	64	76.8 ± 8.8	6–60	1, 3, 6	8
Lu et al. [20]	China	2012	Retrospective observational studies	155	43	73.3 ± 9.8	< 24	5	7
Mo et al. [21]	China	2020	Retrospective observational studies	168	56	–	1–3	2	6
Ren et al. [22]	China	2015	Retrospective observational studies	182	21	49–91	24–50	2	7
Yang et al. [23]	China	2019	Retrospective observational studies	170	10	45.66 ± 1.33	3–13	7, 8	8
Sun et al. [24]	China	2016	Retrospective observational studies	175	37	70.3 ± 8.2	12	4, 5, 6	9

### Data synthesis and analysis

The data from the qualified studies were analyzed using Review Manager 5.3 software provided by the Cochrane Collaboration. The statistical data were analyzed by OR. We used the inconsistency index (I^2^) statistic to evaluate the magnitude of heterogeneity (low heterogeneity, 0–25%; moderate heterogeneity, 25–50%; high heterogeneity, 50–100%). If I^2^ > 50%, we would use a random-effects model; alternatively, we selected a fixed-effect model [25–27]. When the heterogeneity I^2^ > 50%, we would also perform a sensitivity analysis to identify possible reasons for heterogeneity. Sensitivity analysis was performed using the trim and fill method to detect the effects of publication bias on results [27]. Egger's test was used to evaluate publication bias. *P* < 0.05 was considered statistically significant.

## Results

**Fig. 1:**
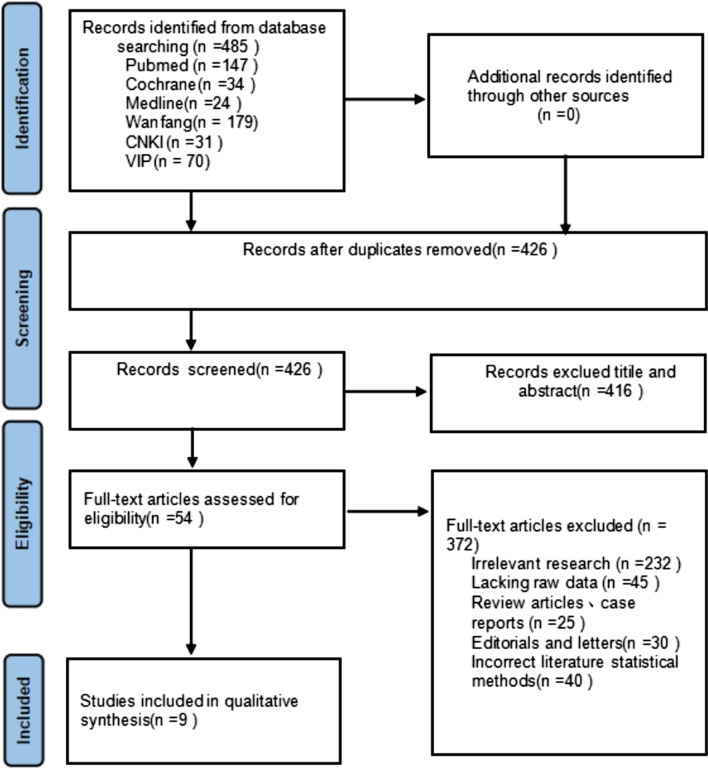
Summary of the evidence search and selection process

A total of 485 literatures were retrieved, and 426 literatures were obtained after eliminating the duplicates. A total of 416 papers were obtained after preliminary screening of papers and abstracts, including review, systematic evaluation, meta-analysis and animal experiment. After reading the full text, 54 articles were obtained after eliminating the literature inconsistent with this study. According to the inclusion and exclusion criteria and intensive reading of the full text, 9 articles were finally included, all of which had a NOS score of ≥ 6 (Fig. 1).

### Meta-analyses and sensitivity analyses

**Fig. 2:**
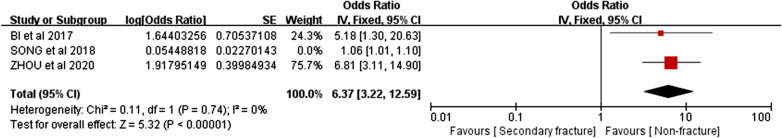
Forest plots for outcomes on the risk of the additional history of fracture; CIs, confidence intervals; OR, odds ratio

Three studies reported the correlation between the additional history of fracture and secondary fractures after percutaneous vertebroplasty for osteoporotic vertebral compression fractures and found a significant difference with the additional history of fracture (OR = 6.37; 95% CI 3.22–12.59; *P* < 0.05; Fig. 2), with low heterogeneity (I^2^ = 0). According to the GRADE approach, the quality of the evidence for the retrospective observational studies data was regarded as high, respectively (Table 2).

**Table 2:** Overall analysis of risk factors for secondary fractures to percutaneous vertebroplasty for osteoporotic vertebral compression fractures

Outcomes	OR	95% CI	*P*	I^2^ (%)	Model
The additional history of fracture	6.37	(3.22, 12.59)	*P* < 0.001	0	Fixed
Age	1.06	(1.03, 1.10)	*P* < 0.001	27	Fixed
BMD	0.25	(0.18, 0.35)	*P* < 0.001	39	Fixed
Bone cement leakage	4.08	(1.18, 14.14)	*P* = 0.03	87	Random
Intravertebral fracture clefts	0.01	(0.00, 0.09)	*P* < 0.001	84	Random
BMI	0.74	(0.23, 2.36)	*P* = 0.61	89	Random
Cobb Angle	0.02	(0.02, 0.03)	*P* < 0.001	99	Random
The height of vertebral anterior	0.48	(0.13, 1.81)	*P* = 0.28	92	Random

**Fig. 3:**
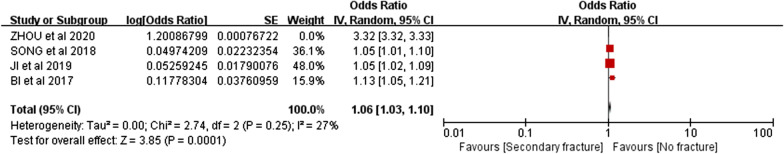
Forest plots for outcomes on the risk of age; CIs, confidence intervals; OR, odds ratio

The risk of age was recorded in four retrospective observational studies. The pooled data significantly favored the risk of age (OR = 3.31; 95% CI 3.31–3.32; *P* < 0.05). However, there was high heterogeneity among these observational studies. Based on the GRADE approach, the quality of the evidence for the retrospective observational studies was regarded as very low, respectively (Table 2). Sensitivity analysis revealed that the study by Zhou et al. [28] was the source of statistical heterogeneity in the meta-analysis for osteolysis. When this outlier study was removed, the 3 remaining studies exhibited low heterogeneity (I^2^ = 27%). Thus, this showed an association between age and secondary fractures after percutaneous vertebroplasty for osteoporotic vertebral compression fractures (OR = 1.16; 95%CI 1.03–1.10; *P* < 0.05; Fig. 3).

**Fig. 4:**
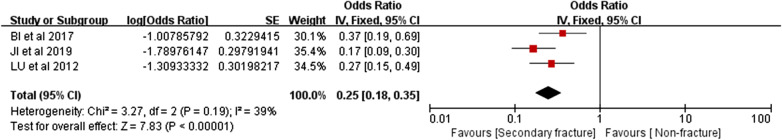
Forest plots for outcomes on the risk of BMD; CIs, confidence intervals; OR, odds ratio

Three studies reported the correlation between BMD and secondary fractures after percutaneous vertebroplasty for osteoporotic vertebral compression fractures and found a significant difference with BMD (OR = 0.25; 95% CI 0.18–0.35; *P* < 0.05; Fig. 4), with low heterogeneity (I^2^ = 39). According to the GRADE approach, the quality of the evidence for the retrospective observational studies data was regarded as high, respectively (Table 2).

**Fig. 5:**
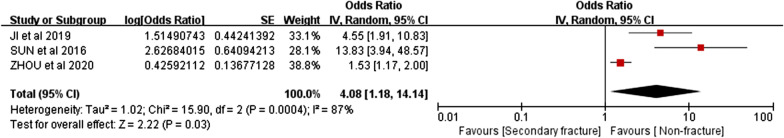
Forest plots for outcomes on the risk of bone cement leakage; CIs, confidence intervals; OR, odds ratio

Three studies reported the correlation between bone cement leakage and secondary fractures after percutaneous vertebroplasty for osteoporotic vertebral compression fractures. There was statistical heterogeneity among the results of the studies, and the random effects model was selected for analysis. Based on the GRADE approach, the quality of the evidence from studies was very low (Table 2). Pooling the data from all studies showed a significant difference (OR = 4.08; 95% CI 1.18–14.14; *P* = 0.03; Fig. 5), but statistically significantly high heterogeneity was observed between the studies (I^2^ = 87%). Sensitivity analysis showed no changes in the high heterogeneity when data from any single trial were removed.

**Fig. 6:**
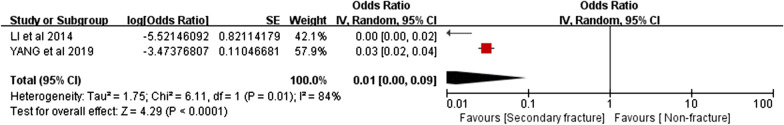
Forest plots for outcomes on the risk of intravertebral fracture clefts; CIs, confidence intervals; OR, odds ratio

Intravertebral fracture clefts was only compared in 2 retrospective observational studies. There was no significant difference between the two groups (OR = 0.01; 95% CI 0.00–0.09; *P* < 0.05; I^2^ = 84%; Fig. 6). The GRADE estimate for quality of evidence was low, resulting from serious imprecision and a high risk of bias (Table 2).

**Fig. 7:**
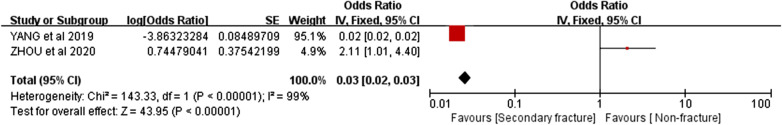
Forest plots for outcomes on the risk of Cobb Angle; CIs, confidence intervals; OR, odds ratio

Cobb angle was only compared in 2 retrospective observational studies. There was no significant difference between the two groups (OR = 0.02; 95% CI 0.02–0.03; *P* < 0.05; I^2^ = 99%; Fig. 7). The GRADE estimate for quality of evidence was low, resulting from serious imprecision and a high risk of bias (Table 2).

**Fig. 8:**
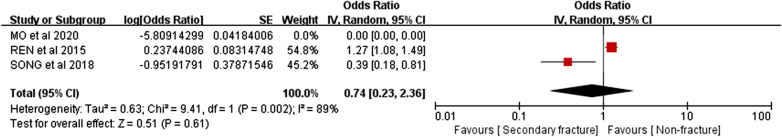
Forest plots for outcomes on the risk of BMI; CIs, confidence intervals; OR, odds ratio

**Fig. 9:**
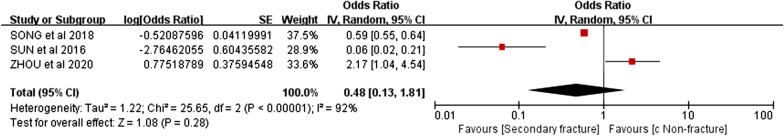
Forest plots for outcomes on the risk of the height of vertebral anterior; CIs, confidence intervals; OR, odds ratio

However, BMI (OR = 0.74; 95% CI 0.23–2.36; *P* = 0.61; I^2^ = 89%; Fig. 8) and the height of vertebral anterior (OR = 0.48; 95% CI 0.13–1.81; *P* = 0.28; I^2^ = 92%; Fig. 9) had no significant difference.

## Discussion

As the population ages, more and more people are suffering from osteoporosis. At present, there are about 200 million osteoporosis patients in the world, and China has the largest number of osteoporosis patients in the world. It is estimated that there will be 4.83 million osteoporotic fractures in China in 2035 and 5.99 million in 2050 [29]. Osteoporotic vertebral compression fracture is one of the most common complications in patients with osteoporosis and has become a global health problem that seriously endangers the health and quality of life of the elderly. As an effective and reliable method for the clinical treatment of symptomatic osteoporotic vertebral compression fracture patients, percutaneous vertebroplasty has been recognized by more and more studies and scholars for its advantages of rapid pain relief, effective improvement of kyphosis and short recovery time. It is not clear whether the recurrence of fracture after percutaneous vertebroplasty is caused by the natural course of osteoporosis or by surgical factors or their own non-surgical factors. It may also be caused by a variety of factors. There have long been studies and reports on the occurrence of postoperative refracture of vertebral compression fractures at home and abroad. Due to the differences in inclusion and exclusion criteria, research methods, surgical personnel skills and postoperative treatment, follow-up time, sample size and other factors, the risk factors obtained from different studies vary greatly, and there are still many controversies. In this study, the latest domestic and foreign studies on postoperative refracture of vertebral compression fractures from January 2011 to January 2021 were collected to comprehensively summarize the relevant factors for secondary fractures after percutaneous vertebroplasty for osteoporotic vertebral compression fractures. The results of the meta-analysis showed that The additional history of fracture, age, BMI, bone cement leakage, Intravertebral fracture clefts and Comb Angle were the risk factors for secondary fractures after percutaneous vertebroplasty for osteoporotic vertebral compression fractures, but body mass index and anterior vertebral height were not related.

Siris et al. [28] found that patients with a history of previous vertebral fractures had an increased risk of vertebral refracture, and the more times of previous vertebral fractures, the greater the probability of refracture. In this study, it was found that the presence of fracture history was associated with the secondary fractures after percutaneous vertebroplasty for osteoporotic vertebral compression fractures. We believe that patients with previous fracture history may already have osteoporosis, and braking after fracture may aggravate the degree of osteoporosis, thereby increasing the risk of refracture.

Advanced age was a robust prognostic factor. Our study reported a significant association between age and refracture after percutaneous vertebroplasty. The previous researchers such as Takahara et al. [30] believed that advanced age was an independent risk factor for refracture after percutaneous vertebroplasty. Tan et al. [31] found in their study that the risk of OVCF in women older than 60 years increased by 1 times for every 5 years. This means that the rate of refracture is higher with age. The literature [32, 33] showed that with the increase of age, the ability to repair damage, the level of sex hormones in the body, and the body's antioxidant capacity gradually decrease. When the body is in a state of oxidative stress, the function of osteoblasts and osteocytes is inhibited, and the function of osteoclasts is enhanced, thus leading to the occurrence of osteoporosis [32], and then vertebral compression fracture. The results of this study showed a significant association between age and refracture after percutaneous vertebroplasty. Generally, as the gerontic patients are the main population receiving PVP, physical status evaluation should be routinely carried out with much attention paid to the advanced age or functional impaired ones.

Bone mineral density (BMD) is an important index to evaluate patients with osteoporosis. With the increase of age, the BMD value of elderly patients decreases, osteoporosis, decreased bone mass, and degeneration of the tough structure of bone occurs. Ma et al. [34] found in a meta-literature analysis that low bone mineral density is a high risk factor for refracture after percutaneous vertebroplasty. In addition, some scholars have found that bone mineral density is closely related to postoperative vertebral refracture, Each 1% increase in BMD was associated with a 3% decrease in the risk of vertebral fracture [35]. Researchers [36–39] found that patients with low bone mineral density significantly increased the risk of refracture. Therefore, we suggest that systematic anti-osteoporosis therapy can significantly reduce the probability of refracture after vertebroplasty.

A large number of studies have found [40, 41] that leakage of bone cement is an influential factor for the occurrence of refractures. During percutaneous vertebral augmentation procedures, when bone cement is injected into the compressed vertebra, it can spill through fractures in the vertebra, especially toward the intervertebral disk [42, 43]. Uppin et al. [37] showed that in OVCF patients with refracture after percutaneous vertebroplasty, about 2/3 of the refracture occurred in the adjacent vertebrae of the affected cone. Studies [37] have shown that leakage of bone cement into the intervertebral space increases the risk of subsequent fractures in adjacent vertebrae, causing severe spinal cord nerve damage and placing a heavy burden on the patient. Mechanism of bone cement leakage leading to refracture of adjacent vertebral body (1) When the bone cement leaks out of the vertebra, especially into the intervertebral space, the stress on the injured vertebral disk is reduced, which will increase the stress on the adjacent vertebral body, and it is easy for the adjacent vertebral body to fracture again. (2) Leakage of bone cement mechanically stimulates the endplate of the adjacent vertebra and accelerates disk degeneration, further increasing the likelihood of refracture of the adjacent vertebra. Therefore, in clinical practice, it is necessary to improve surgical skills and operational norms to reduce the occurrence of bone cement leakage.

Studies [44] have shown that Intravertebral fracture clefts and Cobb Angle are risk factors for secondary fractures after percutaneous vertebroplasty for osteoporotic vertebral compression fractures. Although our meta-pooled results showed that Intravertebral fracture clefts and Cobb angle were statistically significant in the incidence of secondary fractures after percutaneous vertebroplasty for osteoporotic vertebral compression fractures. However, due to the high heterogeneity, it is not possible to prove whether these two risk factors have true evidence-based significance for secondary fractures after percutaneous vertebroplasty for osteoporotic vertebral compression fractures.

However, there were several limitations to this study. First, some articles at home and abroad have reported the influence of other factors on the secondary fractures after percutaneous vertebroplasty for osteoporotic vertebral compression fractures, such as postoperative anti-osteoporosis time, daily sunshine time, lumbar range of motion and other risk factors on the recurrence of fracture after vertebroplasty. However, due to the lack of data or other reasons, it could not be included in this meta-analysis, which had a certain impact on the quality of the analysis and the results of the study. Therefore, its conclusions still need to be verified by literatures with larger sample size and higher quality in the later stage. Third, due to the limited number of studies, the heterogeneity could not always be adequately explored.

Our study had several strengths. First, we presented 8 potential risk factors among which 6 were statistically significant, and the risk factors were summarized in a systematic review, which meant this is by far the first study to quantitatively summarize the risk factors for secondary fractures to percutaneous vertebroplasty for osteoporotic vertebral compression fractures. Second, all the 9 included literatures were retrospective case–control studies with no low-quality reports, and the sensitivity analysis was basically reliable without obvious publication bias. Third, every included study was carefully screened with methodology assessment resulting in a moderate to high quality, which meant the extracted data was reliable.

## Conclusion

In summary, this meta-analysis found secondary fractures exits after percutaneous vertebroplasty for osteoporotic vertebral compression fractures. And we identified 6 significant risk factors from patient-related and operation-related fields. Future efforts should be made to determine which risk factors should be paid more attention to and how to quantify them. Furthermore, clinicians should have a better knowledge of translational medicine and be better at identifying problems in the clinic and actually make it is a critical need for ''bedside to bench to bedside. It is a repeating loop of research-based medical care, in which clinical observations stimulate research (bench), which leads back to the bedside for implementation and further clinical discovery. This meta-analysis suggested surgeons perform cognitive assessment preoperatively and investigated the reasons for the secondary fractures to percutaneous vertebroplasty for osteoporotic vertebral compression fractures. In order to better avoid the occurrence of complications, clinicians should take more precautions during the perioperative period, which requires clinicians to achieve rich surgical skills and clinical experience through a relatively long learning curve and reduce the occurrence of complications.

## Data Availability

The authors declare that all the data supporting the findings of this study are available within the article and its supplementary information files. MY registration number is INPLASY202140128 and the DOI number is 10.37766/inplasy2021.4.0128.

## Abbreviations

PVP: Percutaneous vertebroplasty
BMD: Bone mineral density
BMI: Body Mass Index
VCFs: Vertebral compression fractures
OVCF: Osteoporotic vertebral compression fracture
PRISMA: Preferred reporting items for systematic reviews and meta-analyses
NOS: Newcastle–Ottawa Scale
